# Ultrahigh electromechanical response from competing ferroic orders

**DOI:** 10.1038/s41586-024-07917-9

**Published:** 2024-09-11

**Authors:** Baichen Lin, Khuong Phuong Ong, Tiannan Yang, Qibin Zeng, Hui Kim Hui, Zhen Ye, Celine Sim, Zhihao Yen, Ping Yang, Yanxin Dou, Xiaolong Li, Xingyu Gao, Chee Kiang Ivan Tan, Zhi Shiuh Lim, Shengwei Zeng, Tiancheng Luo, Jinlong Xu, Xin Tong, Patrick Wen Feng Li, Minqin Ren, Kaiyang Zeng, Chengliang Sun, Seeram Ramakrishna, Mark B. H. Breese, Chris Boothroyd, Chengkuo Lee, David J. Singh, Yeng Ming Lam, Huajun Liu

**Affiliations:** 1 Institute of Materials Research and Engineering (IMRE), Agency for Science, Technology and Research (A*STAR), Singapore, Republic of Singapore; 2 School of Materials Science and Engineering, Nanyang Technological University, Singapore, Republic of Singapore; 3 Institute of High Performance Computing (IHPC), Agency for Science, Technology and Research (A*STAR), Singapore, Republic of Singapore; 4 Interdisciplinary Research Center, School of Mechanical Engineering, Shanghai Jiao Tong University, Shanghai, China; 5 Department of Mechanical Engineering, National University of Singapore, Singapore, Republic of Singapore; 6 Singapore Synchrotron Light Source (SSLS), National University of Singapore, Singapore, Republic of Singapore; 7 Centre for Ion Beam Applications, Department of Physics, National University of Singapore, Singapore, Republic of Singapore; 8 Shanghai Synchrotron Radiation Facility (SSRF), Shanghai Advanced Research Institute, Chinese Academy of Sciences, Shanghai, China; 9 Department of Electrical and Computer Engineering, National University of Singapore, Singapore, Republic of Singapore; 10 Institute of Technological Sciences, Wuhan University, Wuhan, China; 11 Facility for Analysis, Characterisation, Testing and Simulation (FACTS), Nanyang Technological University, Singapore, Republic of Singapore; 12 Department of Physics and Astronomy, University of Missouri, Columbia, MO USA

**Keywords:** Ferroelectrics and multiferroics, Electronic devices

## Abstract

Materials with electromechanical coupling are essential for transducers and acoustic devices as reversible converters between mechanical and electrical energy^1–6^. High electromechanical responses are typically found in materials with strong structural instabilities, conventionally achieved by two strategies—morphotropic phase boundaries^7^ and nanoscale structural heterogeneity^8^. Here we demonstrate a different strategy to accomplish ultrahigh electromechanical response by inducing extreme structural instability from competing antiferroelectric and ferroelectric orders. Guided by the phase diagram and theoretical calculations, we designed the coexistence of antiferroelectric orthorhombic and ferroelectric rhombohedral phases in sodium niobate thin films. These films show effective piezoelectric coefficients above 5,000 pm V^−1^ because of electric-field-induced antiferroelectric–ferroelectric phase transitions. Our results provide a general approach to design and exploit antiferroelectric materials for electromechanical devices.

## Abstract (web summary)

Ultrahigh electromechanical response is accomplished by inducing extreme structural instability from competing antiferroelectric and ferroelectric orders.

## Main

Materials with electromechanical coupling, such as piezoelectrics, provide natural transduction between mechanical and electrical information and energy^9,10^. They have thus been widely used as key functional components in wireless communications, acoustic transducers and ultrasonic imaging^1–3^. Under an applied electric field, all materials regardless of symmetry change shape or develop strains because of electrostriction. However, the strain induced by the electrostriction is typically small when compared with that induced by piezoelectricity^11^. Especially, as a sub-category of piezoelectric materials, ferroelectric materials with perovskite structures are the main material system that exhibits large piezoelectric coefficients^8,12,13^. Non-perovskite ferroelectrics such as HfO_2_–ZrO_2_ have also been widely investigated recently because of field-tuneable piezoelectric property^14^ and ultrahigh electrostatic energy storage density^15^.

**Fig. 1:**
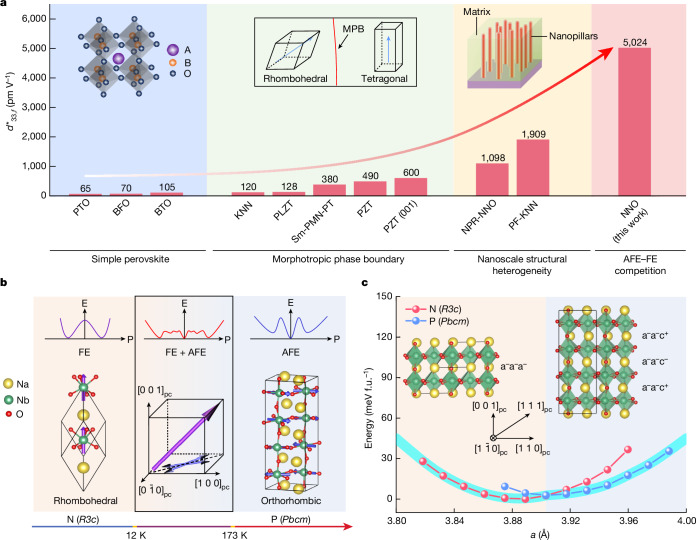
Strategies to enhance the electromechanical response in thin films. **a**, Effective piezoelectric coefficients ${d}_{33,f}^{* }$ of representative thin films from each design strategy (Extended Data Table 1). PTO, PbTiO_3_; BFO, BiFeO_3_; BTO, BaTiO_3_; KNN, K_0.5_Na_0.5_NbO_3_; PLZT, (Pb_0.94_La_0.04_)(Zr_0.6_Ti_0.4_)O_3_; Sm-PMN-PT, Sm-PbMg_1/3_Nb_2/3_O_3_-PbTiO_3_ (71/29); PZT, PbZr_0.52_Ti_0.48_O_3_; PZT (001), (001)-oriented PbZr_0.52_Ti_0.48_O_3_; NPR-NNO, NaNbO_3_ with nanopillar regions; PF-KNN, (K,Na)NbO_3_ with planar faults. AFE, antiferroelectric; and FE, ferroelectric. **b**, Phase transition of NNO from FE N phase (rhombohedral *R3c*) to AFE P phase *(*orthorhombic *Pbcm*) as temperature increases. FE and AFE phases coexist in bulk NNO between 12 K and 173 K. The purple and blue arrows, respectively, represent the polarization directions of the N and P phases. The top panel shows the schematics of Landau energy versus polarization in FE (left), FE and AFE coexistence (middle) and AFE (right) phases. **c**, Calculated free energy as a function of lattice constants (in pseudocubic lattice) for N and P phase NNO. Note that the intersection is located at about 3.9 Å, which guides us to choose SrTiO_3_ (STO) as the substrate for epitaxial growth. The insets show the schematics of crystal structures and octahedral rotations (Glazer’s notations) of NNO *R3c* and *Pbcm* phases. f.u., formula unit.

To effectively integrate into microelectronics, thin films with high electromechanical coupling are in great demand for devices such as microelectromechanical systems (MEMS)^6,16^. We summarized the main strategies to improve the effective piezoelectric coefficient ${d}_{33,f}^{* }$ of thin films in Fig. 1a. As the starting point, simple perovskites have been extensively investigated but show only limited ${d}_{33,f}^{* }$ up to 105 pm V^−1^ (refs. ^17–19^). Structural instability has been identified to play an important part in enhancing electromechanical response, as a large change of structure and strain can be induced by a small electric field in materials with competing structures or phases^20,21^. Two main strategies to induce such structural instability have been widely used in ferroelectric materials. The first strategy is to tune the composition of perovskite ferroelectric oxides to the morphotropic phase boundaries (MPBs), in which two or more ferroelectric phases with different crystal structures are of similar free energy, resulting in a high degree of structural instability and low energy barrier between different phases^7,22^. The representative material systems under this strategy are Pb(Zr,Ti)O_3_ (PZT), (K, Na)NbO_3_ (KNN) and Pb(Mg,Nb)O_3_-PbTiO_3_ (PMN-PT), for which ${d}_{33,f}^{* }$ can be increased up to 600 pm V^−1^ in thin films^23–27^ (Fig. 1a). The second strategy to induce structural instability is by creating nanoscale structural heterogeneity. In bulk ceramics and single crystals, nanoscale heterogeneity is formed by extensive chemical doping to promote the competition between bulk energy and interfacial energy, which greatly improves the piezoelectric properties^13,22,28,29^. In thin films, by using functional defects, self-assembled nanopillar structures with local chemical and structural heterogeneities have shown large ${d}_{33,f}^{* }$ above 1,000 pm V^−1^ (refs. ^8,30^) (Fig. 1a).

**Extended Data Fig. 1:**
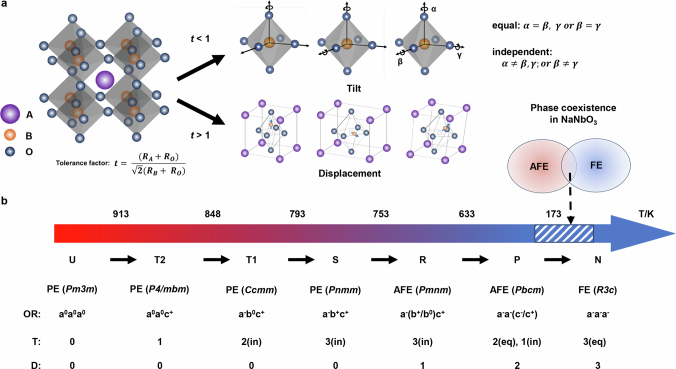
The role of the interaction between octahedral tilt and B-site ionic displacements in the phase diagram of sodium niobate. (a) The schematic of perovskite structure and the preferable behaviour of ionic displacements (*t* > 1) or oxygen octahedral rotation (*t* < 1) in terms of the tolerance factor *t*. The tilt will be considered equal when the tilting angle is equal, while the tilt is independent when the tilt angle is different. (b) Phase diagram of sodium niobate as a function of temperature, where D represents the displacement and T represents the tilts. The notation *eq* and *in* represent the equal and independent respectively. The independent tilting promotes the stabilization of the antiferroelectric phase. The ionic displacements and octahedral rotations induce the competition of ferroelectric and antiferroelectric orders in sodium niobate.

In this work, we demonstrate a different strategy by introducing structural instability from competing antiferroelectric (AFE) and ferroelectric (FE) phases in sodium niobate (NaNbO_3_, NNO) thin films, achieving ultrahigh ${d}_{33,f}^{* }$ above 5,000 pm V^−1^ (Fig. 1a). AFE materials have antiparallel polarizations that are cancelled and show zero macroscopic polarization^31,32^. An intrinsic feature of AFE materials is the existence of a closely related FE phase with slightly higher energy than that of the AFE phase^33,34^. Under the applied electric field, the energy of this FE phase is lowered such that AFE–FE phase transitions are induced, typically with large strain and high piezoelectric coefficients^35^. Guided by the bulk phase diagram of NNO and theoretical calculations, we designed the coexistence of AFE and FE NNO phases by stabilizing the low-temperature FE phase. NNO has a complex phase diagram with seven crystalline phases, including paraelectric (PE), AFE and FE phases^36^ (Extended Data Fig. 1). At room temperature, NNO shows orthorhombic AFE phase (P phase with space group *Pbcm*). The FE NNO phase (N phase with space group *R3c*) occurs at temperatures lower than 173 K. Recent investigations of bulk NNO shows the coexistence of FE and AFE phases in the temperature range between 12 K and 173 K (refs. ^36–40^) (Fig. 1b). With the coexistence of AFE and FE phases, a high degree of flexibility in polarization orientations is possible from the reported relaxor behaviour of NNO in this temperature range^41^. The polarization directions of AFE and FE phases are shown in pseudocubic unit cell (Fig. 1b, middle). From the aspect of thermodynamic energy, the flexibility in polarization also contributes to the flattening of Landau energy profile (Fig. 1b, top), giving rise to a drastic enhancement in the piezoelectric coefficient^22^. Inspired by this phase coexistence, we performed the first-principles calculations to understand the free energy profiles of FE and AFE NNO phases. These two phases show small energy differences depending on the lattice dimensions. Especially, the energy curves overlap at the lattice dimension of about 3.9 Å (Fig. 1c), which is very close to the lattice parameter of SrTiO_3_ (STO) single crystal substrates. Therefore, we choose STO substrates to construct the phase coexistence of AFE and FE phases for NNO epitaxial thin films. Moreover, as bulk FE N phase is stable only at temperatures below 173 K, it is challenging to stabilize it at room temperature. Because the FE N phase has a rhombohedral symmetry, the three-fold symmetry from (111) crystal orientation could help to constrain the lattice symmetry and stabilize the N phase in the thin films at room temperature. We thus select STO substrates in (111) orientation for our NNO film growth. A close check of the atomic structures shows similar atomic arrangements of FE and AFE phases with a subtle shift of atomic positions and different octahedral rotation patterns (Fig. 1c (inset) and Supplementary Fig. 1).

## Structure of NNO films

**Fig. 2:**
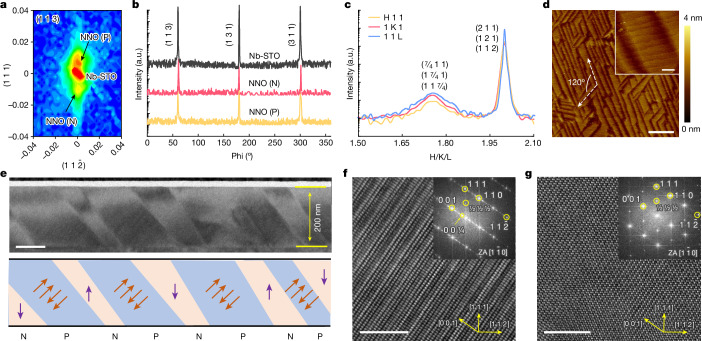
Crystal structure and surface morphology of the 200-nm-thick NNO films. **a**–**c**, Synchrotron X-ray based (113) reciprocal space mapping (**a**), phi scan (**b**) and characterization of 7/4 diffraction peaks by H/K/L scan (**c**) of the NNO/Nb-STO (111) heterostructure. The pseudocubic coordinate system is used for convenience. **d**, Surface morphology of the NNO film using an atomic force microscope. The RMS roughness is about 300 pm. The white dashed lines show the angle between two domains from the top view. The inset shows the fine terrace features. The colour bar represents the height. **e**, Low-magnification cross-sectional bright-field TEM image (top) and schematic of phase coexistence (bottom) of NNO/Nb-STO (111) heterostructure along the zone axis $[1\bar{1}0]$. The arrows represent the directions of polarization. **f**,**g**, High-resolution TEM images of the P phase (**f**) and N phase (**g**) along the zone axis $[1\bar{1}0]$. The insets show their respective indexed FFT patterns, demonstrating different crystal symmetries. a.u., arbitrary units. Scale bars, 2 μm (**d**); 200 nm (**d**, inset); 100 nm (**e**); 5 nm (**f**,**g**).

**Extended Data Fig. 2:**
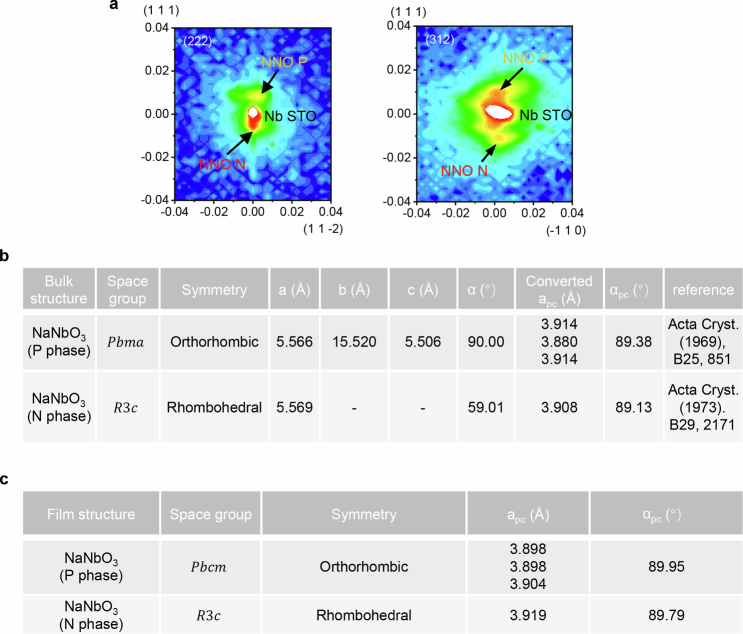
Structural characterization of the 200 nm-thick NNO films. (a) Reciprocal space mapping of the NNO film along the (222) and (312) reflections. (b) Structure information of bulk NNO P and N phase^40,64^. (c) Calculated structure parameters of the NNO film based on the RSV method^42^.

**Extended Data Fig. 3:**
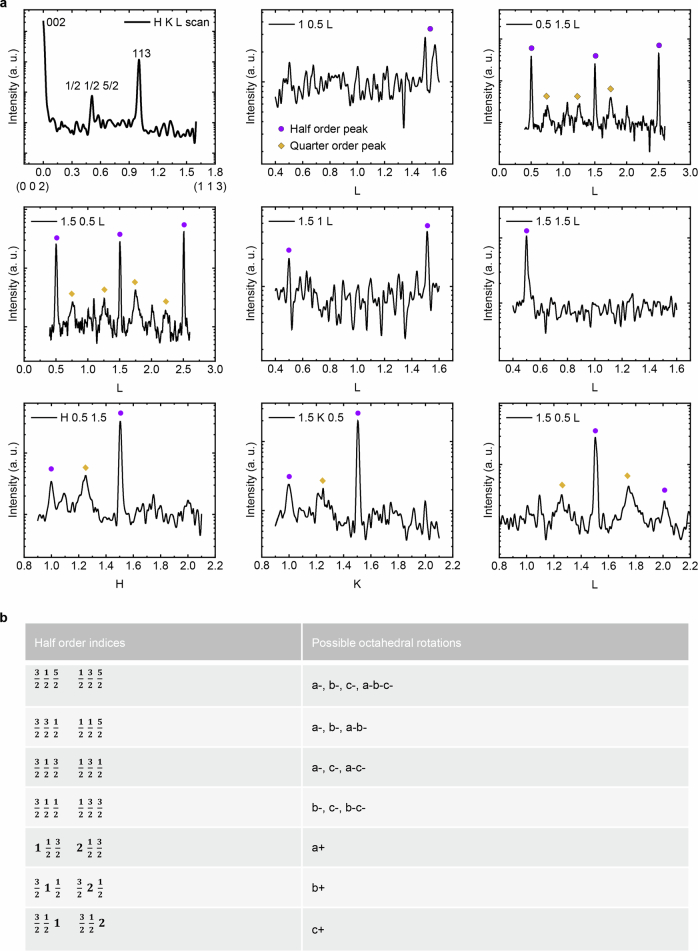
Oxygen octahedral rotation behaviour in the 200 nm-thick NNO films. (a) Half order diffraction characterization of the NNO film. (b) Summarized oxygen octahedral rotation behaviors in the NNO film based on the combination of the half order diffraction peaks.

**Extended Data Fig. 4:**
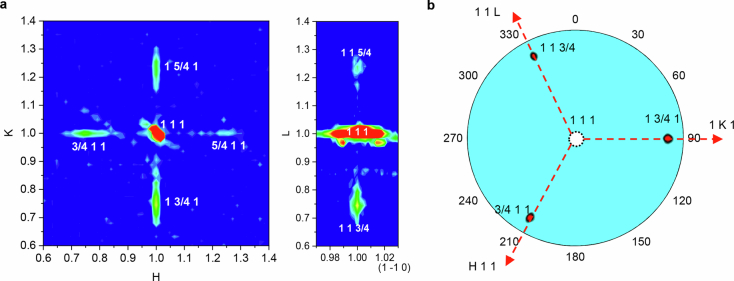
RSM of quarter order diffractions of the 200 nm-thick NNO films. (a) HK mapping (left) and ($[1\bar{1}0]$)L mapping (right) around (111) diffraction peaks of substrate, showing quarter-order diffraction peaks along all H, K, L directions. (b) A three-fold symmetry in RSM of (1 1 3/4), (1 3/4 1) and (3/4 1 1) diffractions as a result of the arrangement of the P phase domain along H, K, and L directions.

We grew 200-nm-thick epitaxial NNO films on the (111)-oriented Nb-doped STO (Nb-STO) single crystal substrates (Methods and Supplementary Fig. 2). Using the synchrotron-based X-ray diffraction (XRD), the NNO N and P phases show the (444)_R_ and (048)_O_ diffraction peaks (both are (222) in pseudocubic notation) around the (222) diffraction peak of the substrate (Supplementary Fig. 3). The pseudocubic coordinate system is used in the following discussions. We performed the reciprocal space mappings (RSMs) of the NNO/Nb-STO heterostructure (Fig. 2a). The (113) RSM indicates that N and P phases are epitaxially grown on the substrate in a coherently strained state. Combining with the other two independent RSMs of the (222) and (312) planes (Extended Data Fig. 2), these two phases show a rhombohedral and orthorhombic symmetry, respectively. The calculated lattice parameters are *a*_*pc,N*_ = 3.919 Å for the N phase and *a*_*pc,P*_ = 3.898 Å for the P phase using the reciprocal space vector method^42^. The N phase has a *R3c* structure and the P phase has a structure of *Pbcm* based on our diffraction data, consistent with previous studies^36,43^. Moreover, the estimated ratio of the N phase and P phase is about 1:3 (Supplementary Fig. 4). We conducted the XRD φ (Phi)-scans for the (113) peak of Nb-STO, NNO N and P phases to confirm the epitaxial relationship (Fig. 2b). The result shows the global three-fold symmetry of both N and P phases. Furthermore, our half-order diffraction scan results show that the sample contains all types of oxygen octahedral rotation behaviour (that is, ${a}^{\pm }{b}^{\pm }{c}^{\pm }$) in Glazer’s notations (Extended Data Fig. 3), suggesting that the three-fold symmetry of the orthorhombic phase may be attributed to the arrangement of the structural domains. Figure 2c shows the XRD line scans of the quarter diffraction peaks of the P phase along the (H 1 1), (1 K 1) and (1 1 L). The appearance of (7/4 1 1), (1 7/4 1) and (1 1 7/4) diffraction peaks along three mutual orthogonal scans confirms the three-fold symmetry of arrangements of the P phase. Besides, the similar diffraction intensity of these quarter peaks implies that the domains have almost equal distribution along three directions. The quarter diffractions resulting from the antipolar displacement in the P phase and the three-fold symmetry caused by the P phase arrangement are also confirmed by RSMs (Extended Data Fig. 4). We scanned the surface morphology of the NNO film using an atomic force microscopy (AFM) (Fig. 2d). The NNO P phase (bright contrast) shows an accurate 120° angle relationship of domains from the top view, matching with the three-fold symmetry from the XRD data. It also indicates that the N phase (dark contrast) sits in between the P phase domains. Furthermore, the film has a smooth surface with a clear unit-cell terrace structure over an area of 1 × 1 μm^2^ and a root-mean-square (RMS) roughness of ~ 300 pm as shown in Fig. 2d (inset), indicating a high film quality.

**Extended Data Fig. 5:**
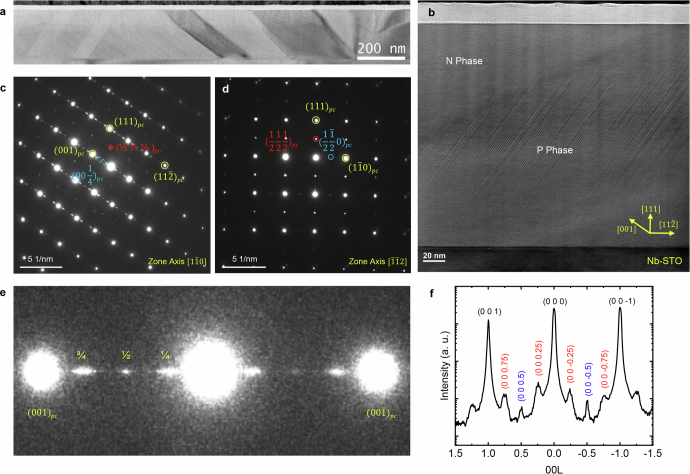
TEM images and SAED patterns of the 200 nm-thick NNO films. (a) Low magnification cross-sectional TEM images were taken along ZA $[\bar{1}\bar{1}2]$. (b) N phase and P phase boundary image. The P phase shows clear dark stripes that are perpendicular to the [001] direction compared to the N phase. These regular stripes indicate the AFE modulation of the NNO P phase. Indexed selected area electron diffraction (SAED) patterns taken along (c) ZA $[1\bar{1}0]$ and (d) ZA $[\bar{1}\bar{1}2]$. Only one set of main diffraction spots was demonstrated, suggesting that the N and P phases have very similar symmetry and lattice. The superlattice indices including half and quarter reflections indicate that there may be some modulation like oxygen octahedral rotation or atomic anti-ferroelectric arrangement in the NNO film. (e) Extracted quarter diffraction peaks from the SAED pattern. (f) Intensity profile image of figure e.

**Extended Data Fig. 6:**
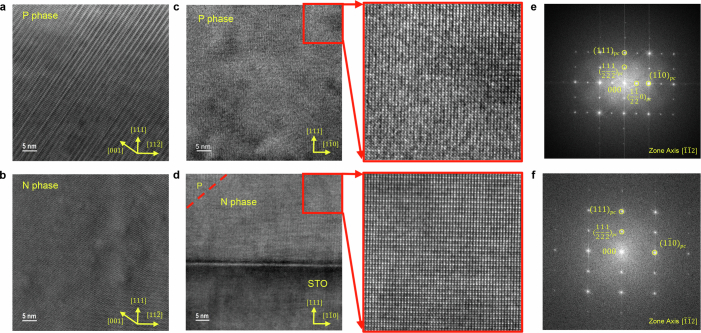
HR-TEM images and diffractograms from the 200 nm-thick NNO films. (a, b) HR-TEM images of (a) P phase and (b) N phase taken along ZA$[1\bar{1}0]$. (c, d) HR-TEM images of (c) P phase and (d) N phase taken along ZA$[\bar{1}\bar{1}2]$ and (e, f) their corresponding indexed FFT patterns. The red square shows the respective enlargement images of P and N phases.

**Extended Data Fig. 7:**
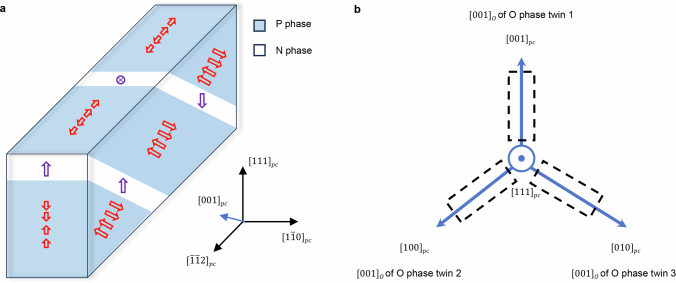
Schematics of domain arrangement of the N and P phase in the 200 nm-thick NNO films. (a) Schematic drawing of the domain structures of twin 1 in the NNO film. The AFE phases are separated by the FE phase and the FE phase has polarization along the out-of-the-plane direction (up or down), while the AFE phase shows pairs of spontaneous polarizations with an inclined polarization direction. (b) Schematic drawing of three-fold symmetric domain arrangement from the [111] projection view. The P phase are formed in the directions of [001], [010], and [100], resulting in three-fold symmetry, agreeing with the symmetry modulation from the (111) oriented substrate.

To further investigate the crystal structure, we performed electron microscopy characterizations along two orthogonal directions (zone axis (ZA) $[\bar{1}\bar{1}2]$ and ZA $[1\bar{1}0]$). A low-magnification cross-sectional transmission electron microscopy (TEM) image shows the mixture of the N and P phases (Fig. 2e, top), which are consistent with the surface morphology. We show a schematic arrangement of the AFE and FE phases and their corresponding polarization directions in Fig. 2e (bottom). The coexistence of the N and P phases is further confirmed in terms of the difference in atomic arrangement and electron diffraction patterns (Extended Data Fig. 5). We took high-resolution TEM (HRTEM) images and their fast Fourier transforms (FFTs) in the single domain areas to further analyse the structure of the N and P phases (Fig. 2f,g and Extended Data Fig. 6). The quarter reflections shown in the P phase are consistent with our XRD results. The half-index reflections demonstrate the oxygen octahedral rotation behaviour: the 1/2 (odd odd odd) and 1/2 (odd odd even) reflections in the P phase indicate that the P phase has two kinds of oxygen octahedron rotation modes noted as *a*^*−*^*a*^*−*^*c*^*+*^*/a*^*−*^*a*^*−*^*c*^*−*^, which contribute to the quarter reflections along the [001]. The N phase shows only the 1/2 (odd odd odd) reflections, indicating that only the *a*^*−*^*a*^*−*^*a*^*−*^ pattern exists. High-angle annular dark field images taken by scanning transmission electron microscopy show the similar atomic structure of the N and P phases, which could be distinguished by diffraction patterns (Supplementary Figs. 5 and 6). The schematic was drawn to describe the phases coexistence (Extended Data Fig. 7).

## Ferroic properties and electromechanical response

**Fig. 3:**
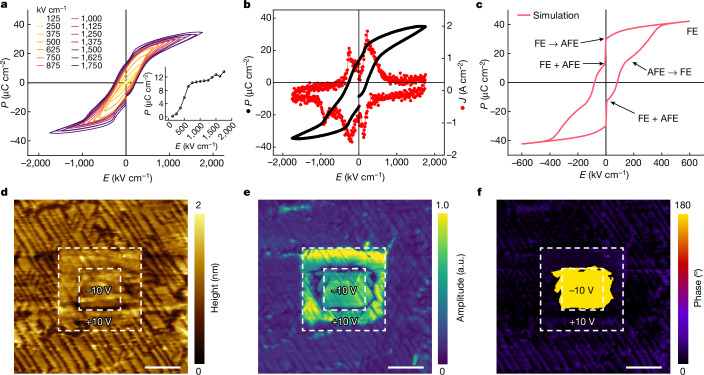
Antiferroelectric and ferroelectric behaviour of the 200-nm-thick NNO films. **a**, Electric-field-dependent polarization curves of the NNO film. The inset shows the remnant polarization as a function of the applied electric field. **b**, Dynamic ferroelectric hysteresis loop and corresponding switching current curve of the NNO film. **c**, Simulation of the ferroelectric hysteresis loop of FE and AFE coexistence with a ratio of 1:3 based on Landau theory. **d**–**f**, PFM images of topography (**d**), amplitude (**e**) and phase (**f**) measured after writing the FE domain. a.u., arbitrary units. Scale bar, 2 μm (**d**,**e**,**f**).

**Extended Data Fig. 8:**
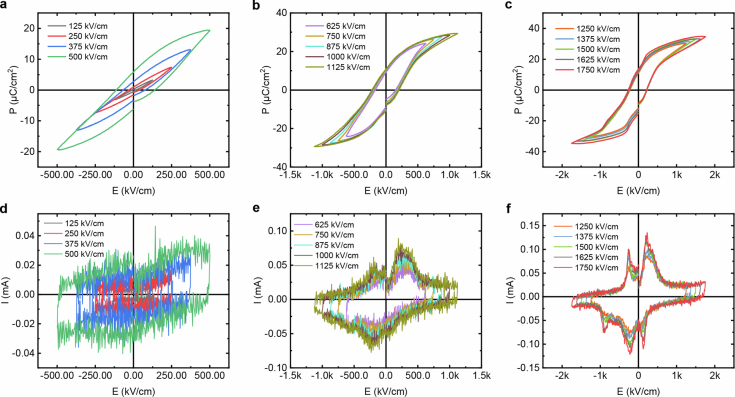
Ferroelectric characterization of the 200 nm-thick NNO films. (a-c) P-E loops of (a) 125–500 kV/cm, (b) 625–1125 kV/cm, (c) 1250–1750 kV/cm, and (d-f) corresponding switching current curves: (d) 125–500 kV/cm, (e) 625–1125 kV/cm, (f) 1250–1750 kV/cm.

To further confirm the FE and AFE behaviour of the NNO film, we measured the polarization-electric field (P–E) loops and corresponding switching curves (Fig. 3a and Extended Data Fig. 8). Owing to the initial N phase, the NNO shows a FE nature when the electric field is too small to trigger the phase transition of the AFE phase. As shown in Fig. 3a (inset), the NNO films show a maximum remnant polarization *P*_r_ of about 13.6 μC cm^−2^, which is attributed to the FE phase, agreeing with the result measured by PUND (positive up negative down) configuration (Supplementary Fig. 7). We observed the AFE–FE phase transition from the changes of the P–E loop and corresponding current switching curve with five current switching peaks (Fig. 3b). The saturation polarization reaches a polarization value of about 34.6 μC cm^−2^ at an electric field of 1,750 kV cm^−1^ and the coercive electric field is around 210 kV cm^−1^. Electric field higher than 1,750 kV cm^−1^ results in an electric breakdown in the NNO thin film. We simulated the P–E loop of NNO film with an FE:AFE ratio of 1:3 by the Landau theory. The results indicate a process that the NNO film goes through a phase transition from AFE and FE coexistence to pure FE phase at a high electric field (Fig. 3c). Although the P–E loops show FE behaviour, no obvious intrinsic FE domain distribution can be observed in both out-of-plane and in-plane phase contrast (Supplementary Fig. 8) using a piezoresponse force microscopy (PFM). This could be because the dominant phase is the AFE phase in the NNO film. We also used a PFM tip to write, switch and read the FE domains of the NNO film (Fig. 3d–f). The clear edge in amplitude and contrast in the phase image indicates that the FE domain can be written, and the FE domain can be switched using an external bias of 10 V. The extended domain region beyond the switching area indicates that the domain switching could be driven by switching activity in the neighbouring domains (Fig. 3f). The domain writing and switching properties confirm the AFE–FE phase transition under the applied electric field.

**Fig. 4:**
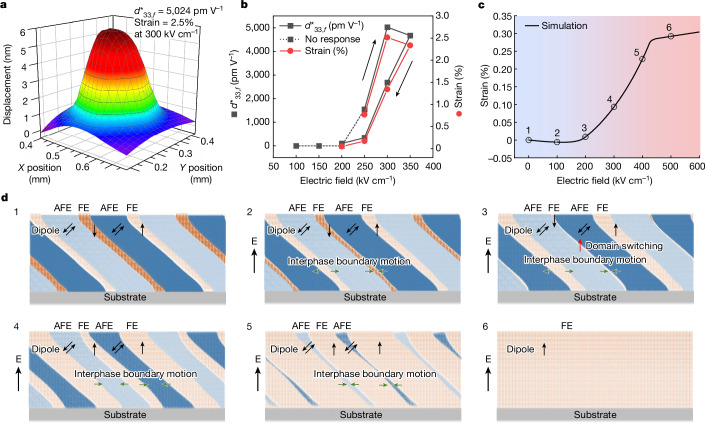
Electromechanical response of the 200-nm-thick NNO films and simulation of domain dynamics during AFE–FE phase transition. **a**, Three-dimensional mapping of surface displacement of the NNO film measured under a.c. drive amplitude of 1 V at 1 kHz. **b**, Electric-field-dependent effective piezoelectric coefficient and strain of the NNO film at 1 KHz. **c**,**d**, Evolution of strain change (**c**) and domain switching and interphase boundary motion (**d**) in the NNO film under various electric fields based on the phase-field simulation. The dynamics of dipoles (black arrows in **d**) responsive to an electric field manifests itself as a motion of FE/AFE interfaces followed by a domain switching (red arrows) and finally a transformation into a pure FE phase.

**Extended Data Fig. 9:**
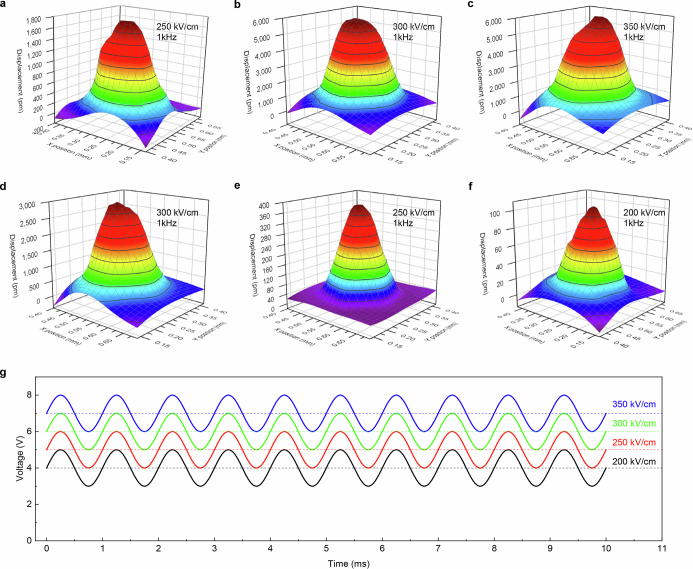
Electromechanical response of the 200 nm-thick NNO films. (a-f) External electric field-dependent surface displacement measured by laser Doppler vibrometer with an a.c. voltage of 1 V at a frequency of 1 kHz. (g) Inducing external electric field by applying d.c. voltage during laser Doppler vibrometer test (only show 10 ms data).

We investigated the electromechanical response of the NNO films by measuring the surface displacements using a laser Doppler vibrometer with an a.c. (alternating current) drive of 1 V in amplitude at 1 kHz (Fig. 4a and Extended Data Fig. 9). Under an applied electric field of 300 kV cm^−1^, the effective piezoelectric coefficient ${d}_{33,f}^{* }$ is about 5,024 pm V^−1^ and the electric-field-induced strain is around 2.5%. The NNO films show a nonlinear behaviour with an enhanced strain as the electric field increased (Fig. 4b). A small strain hysteresis was observed in the NNO film, which is different from the point-defect-induced strong hysteretic behaviour in aged BaTiO_3_ (ref. ^44^), indicating a phase-transition-induced strain under an external electric field. Frequency-dependent measurements show that the displacement increases as the frequency decreases and the effective piezoelectric coefficient could reach a magnitude of 18,400 pm V^−1^ with a strain of 9.2% at a low frequency of 200 Hz (Supplementary Fig. 9). No mechanical failures such as cracks or delamination were observed in samples after vibrometer testing (Supplementary Fig. 10). The giant strain at low frequency mainly arises from domain walls or interphase boundaries motion under electric field, whereas possible electrochemical effects such as the electric-field-induced rearrangement of defects^45^ or Schottky barrier^5^ may also contribute to the electromechanical response.

The total electromechanical response in ferroelectrics could be considered as a combination of spontaneous polarization change (intrinsic) and domain/interphase wall motion (extrinsic). To better understand the domain dynamics and the subsequent strain response under an electric field along the out-of-plane direction, we performed phase-field simulation for the NNO film with coexistence of AFE and FE phases. As shown in Fig. 4c, the qualitative trend of strain change is consistent with our electric-field-dependent piezoelectric response measurements and the evolution of the P–E loop as well. Figure 4d shows the corresponding domain movement dynamics. In the initial state, AFE and FE coexisted in the thin film in which the FE phase has domains with upward and downward polarizations. When an upward electric field is applied, the FE domains with an upward dipole extend; the FE domains with a downward polarization first reduce in size till they almost fully disappear and then switch to form new domains with an upward polarization aligning parallel to the electric field. The polarization rotation occurs at the interphase boundaries to promote the motion of interphase boundaries. However, during this process, the strain response is low because of the dominant portion of the AFE phase. The AFE phase then starts to transform into the FE phase when the electric field is further increased until the whole film changes to a single FE domain with polarization parallel to the applied electric field. The strain changes greatly during this process, indicating the vital role of phase transition from AFE to FE phase. We also simulated the piezoelectric coefficient distribution in the NNO film under the electrical field that induces AFE–FE phase transition (Supplementary Fig. 11), showing an ultrahigh electromechanical response at the interphase boundary. Moreover, as shown in Fig. 1c, the small energy difference in AFE and FE phases enables a large lattice variation from 3.86 Å to 3.94 Å (about 2%), contributing to the ultrahigh electromechanical response as well.

## Discussion

The piezoelectric coefficient in ferroelectric materials could be described by the formula *d* = *2QεP* (ref. ^31^), where *Q* is the electrostrictive coefficient, *ε* is the dielectric permittivity and *P* is the polarization. The different strategies that induce structural instability aim to enhance the dielectric permittivity by reducing the curvature of the function of the free energy density profile. Our DFT calculation results show that the substrate symmetry constraint induced more polarization freedom in the AFE P phase (Supplementary Fig. 12), reducing the energy barrier of polarization switching (Supplementary Fig. 13), similar to the thermodynamic energy profile in materials at MPB compositions^22^. Furthermore, the field-induced AFE–FE phase transition is always accompanied by a drastic microscopic polarization enhancement. Therefore, both increased dielectric permittivity and polarization could potentially contribute to the ultrahigh effective piezoelectric coefficient in the NNO films.

In summary, we demonstrated the strategy to use AFE–FE phase competition to greatly enhance the electromechanical response in NNO thin films. The construction of AFE and FE phase coexistence in thin films through symmetry engineering could pave the way for designing high-performance electromechanical devices based on AFE materials.

## Methods

### Thin film growth

The conventional solid-state reaction method was used to fabricate the ceramic targets of NaNbO_3_ using Na_2_CO_3_ (99.0%) and Nb_2_O_5_ (99.9%) as the raw materials. The 3-inch target with 10 mol% excess of Na was used in this study. Epitaxial NNO films with 200 nm thickness (Fig. 2e, top) were grown on Nb-doped SrTiO_3_ (111) substrates by sputtering at 790 °C with Ar:O_2_ = 2:1, power of 100 W and a deposition time of 4 h. The Nb-doped SrTiO_3_ substrate (Shinkosha) was cleaned in acetone before putting it into the sputtering chamber. The NNO film has a stoichiometry of Na_1.16_NbO_3.08_ derived from the X-ray photoemission spectroscopy (XPS) and Rutherford backscattering spectroscopy (RBS) measurements (Extended Data Fig. 3).

### Characterization

#### X-ray photoelectron spectroscopy

Surface analysis was performed using XPS Kratos AXIS Supra monochromatic Al-Kα (1486.6 eV) X-ray beam. Charge neutralization was used to correct the charge shift by irradiating low-energy electrons and ion beams onto the sample. XPS narrow scan spectra for Na 1*s*, O 1*s* and Nb 3*d* were collected. The background type used is Shirley. The spectra were processed using CasaXPS v.2.3.23 software (ref. ^46^).

#### Rutherford backscattering spectroscopy

RBS experiments were performed using a nuclear microprobe at the Centre for Ion Beam Applications, National University of Singapore^47^. A 1.5 MeV He^+^ beam was focused to micrometre dimensions by a quadrupole lens system. The count frequency on a silicon surface barrier detector was about 1,500 Hz by controlling the incident beam current. Single measurement continuously proceeds for about 30 min to collect enough signals. The experimental RBS spectrum was fitted by a program named SIMNRA (v.7.02) (ref. ^48^). The experimental settings were validated using a standard WSi sample.

#### Synchrotron X-ray diffraction

The synchrotron-based X-ray diffraction experiments were conducted at room temperature in the X-ray development and demonstration beamline of the Singapore Synchrotron Light Source and the surface diffraction beamline (BL02U2) at Shanghai Synchrotron Radiation Facility. The lattice parameters of the orthorhombic antiferroelectric phase and rhombohedral ferroelectric phase in the NNO film were calculated based on the reciprocal space vector method^42^.

#### Electron microscopy characterizations

A dual beam focused ion beam (FIB) (FEI DB Helios 450 S Nanolab) was used to prepare an ultrathin sample (about 50 nm) for TEM and scanning transmission electron microscopy (STEM) measurements. The specimen cutting was conducted using 30 kV Ga ions, and the specimen cleaning was conducted using 1 kV Ga ions. Cross-sectional and atomic structural images were taken using TEM with a 200-keV field-emission gun and a probe current of 0.5 nA at 1 nm. The instrument used was an FEI Tecnai G2 TF20 TEM. Atomic resolution images were taken using a 200-kV cold field-emission probe-corrected transmission electron microscope (JEM-ARM200F) with a STEM resolution of 0.078 nm. Images were monitored and taken using a Gatan 4,000 × 4,000 one-view CMOS camera. DigitalMicrograph software was used to analyse the TEM and STEM data.

#### Electrical properties

A manual probe station is used for electrical tests. Top electrodes Au/Pd with diameters of about 200 μm were deposited by an e-beam evaporator. Dynamic ferroelectric polarization-electric field loops were measured at an interval of 0.3 ms with top–top geometry using a ferroelectric tester (Precision Multiferroic II, Radiant Technologies). A laser Doppler vibrometer was used to obtain the effective piezoelectric coefficient by measuring the surface displacement on the electrode of the tested thin film under an applied electric field. The electric field consists of a small a.c. voltage with an amplitude of 1 V (*V*_a.c._ = sin *ωt*) and a d.c. bias used for inducing the phase transition. Only the first harmonic strain was measured during the test.

#### PFM measurements

PFM measurements were conducted on a commercial scanning probe microscope system (MFP-3D, Asylum Research) at room temperature under ambient conditions. A Pt-coated conductive probe (240AC-PP, OPUS) with a force constant of around 2 N m^−1^ and free resonance frequency of about 70 kHz was used. During PFM scanning, the piezoresponse signal was amplified by using contact resonance, and the Dual AC Resonance Tracking mode was enabled to keep the resonance tracked. An a.c. drive of 2–8 *V*_pp_ was typically used for PFM mapping. The FE domain writing was performed by firstly scanning a 5 × 5 µm^2^ area with a +10 V tip bias, and then followed by a magnification of scanning (2.5 × 2.5 µm^2^) with a −10 V tip bias.

### First-principles calculations

We studied the structural phase transitions and electronic structures of NaNbO_3_ by using the first-principles calculations within GGA-PBESol approximation^49^ as implemented in the WIEN2K software package, the fully localized limit using full-potential (linearized) augmented plane wave (FP-LAPW) + local orbital method^50^. The simulations have been calculated with the augmented plane wave + local orbital (APW + lo) basis set and the muffin-tin radii (*R*_MT_) for the atomic spheres were chosen as 2.12 a.u., 1.78 a.u. and 1.61 a.u. for Na, Nb and O, respectively. The partial waves were expanded up to *l*_max_ = 10 in the muffin-tin spheres and outside a plane-wave cutoff *K*_max_ defined by *R*_min_*K*_max_ = 8.0. Fourier expansion of the charge density was performed up to *R*_min_*G*_max_ = 20.0. Here, *R*_min_ is the minimum sphere radius, that is, 1.61 a.u. The Na 2*s*^2^2*p*^6^3*s*^1^, Nb 4*s*^2^4*p*^6^4*d*^4^5*s*^1^ and O 2*s*^2^2*p*^4^ were treated as semi-core states and were included in the valence. The Brillouin zone sampling was done using a Kmesh of 10 × 10 × 10, and 10 × 10 × 3 *k*-points in the full Brillouin zone for *R3c* and *Pbcm* structures, respectively. All structures are fully relaxed by using energy minimization with Hellmann–Feynman force of 1 mRy a.u.^−1^. For each strain state, that is, different in-plane lattice constant *a*, lattice constant *c* and all atomic positions were fully relaxed until the final Hellmann–Feynman forces were less than 1 mRy a.u.^−1^. The polarization of *R3c* structure is obtained by the Berry phase simulation, which is implemented in the WIEN2K code^51^, *P* = 6.24 μC cm^−2^, at *a* = 3.889 Å. The schematics of atomic structures demonstrated in this paper were drawn by VESTA software^52^.

### Phase-field simulations

In the phase-field model, the microstructure of the NNO film is described by the ferroelectric polarization field **P**(**x**) and the antiferroelectric order parameter **A**(**x**), where **x** is the spatial position vector. The stable state of the system under given conditions as well as the response of the system to given external fields is simulated by evolving the fields, **P** and **A**, to equilibria as governed by the time-dependent Ginzburg–Landau equation^53,54^, written as

1 $${{\boldsymbol{\gamma }}}_{{\rm{P}}}\frac{\partial {\bf{P}}}{\partial t}=-\frac{\partial F}{\partial {\bf{P}}}+{{\bf{E}}}^{{\rm{P}},{\rm{therm}}}.$$

2 $${{\boldsymbol{\gamma }}}_{{\rm{A}}}\frac{\partial {\bf{A}}}{\partial t}=-\frac{\partial F}{\partial {\bf{A}}}+{{\bf{E}}}^{{\rm{A}},{\rm{therm}}}.$$

Equations (1) and (2) describe a relaxation process of the system driven by a minimization of the free energy of the system under given conditions. Here **γ**_P_ and **γ**_A_ are the damping coefficients for ferroelectric polarization and antiferroelectric order, respectively, *t* is the time, and *F* is the free energy of the system. **E**^P,therm^ and **E**^A,therm^ are random fields arising from thermal fluctuations, which obey a normal distribution with the strength given by the fluctuation–dissipation theorem^54,55^.

The free energy *F* is formulated as a functional of the ferroelectric polarization field, antiferroelectric order parameter and external conditions such as the external electric field **E**^ext^, that is, *F* = *F*[**P**(**x**), **A**(**x**), **E**^ext^], following existing phase-field theories^53^. It takes a sum of the Landau free energy for ferroelectric polarization, the Landau free energy for antiferroelectric order, the coupling energy between ferroelectric polarization and antiferroelectric order, the gradient energy, the electrostatic energy and the elastic energy, that is,

3 $$F={F}_{{\rm{Landau}}}^{{\rm{P}}}+{F}_{{\rm{Landau}}}^{{\rm{A}}}+{F}_{{\rm{coupling}}}+{F}_{{\rm{gradient}}}+{F}_{{\rm{electrostatic}}}+{F}_{{\rm{elastic}}}.$$

The energy contributions are given by

4 $${F}_{{\rm{Landau}}}^{{\rm{P}}}=\int \left({a}_{i}{{P}_{i}}^{2}+{a}_{ij}{{P}_{i}}^{2}{{P}_{j}}^{2}+{a}_{ijk}{{P}_{i}}^{2}{{P}_{j}}^{2}{{P}_{k}}^{2}+{a}_{ijkl}{{P}_{i}}^{2}{{P}_{j}}^{2}{{P}_{k}}^{2}{{P}_{l}}^{2}\right){\rm{d}}{x}^{3},$$

5 $${F}_{{\rm{Landau}}}^{{\rm{A}}}=\int \left({b}_{i}{{A}_{i}}^{2}+{b}_{ij}{{A}_{i}}^{2}{{A}_{j}}^{2}+{b}_{ijk}{{A}_{i}}^{2}{{A}_{j}}^{2}{{A}_{k}}^{2}+{b}_{ijkl}{{A}_{i}}^{2}{{A}_{j}}^{2}{{A}_{k}}^{2}{{A}_{l}}^{2}\right){\rm{d}}{x}^{3},$$

6 $${F}_{{\rm{coupling}}}=\int {d}_{ij}{{P}_{i}}^{2}{{A}_{j}}^{2}{\rm{d}}{x}^{3},$$

7 $${F}_{{\rm{gradient}}}=\int {g}_{ijkl}^{{\rm{P}}}\frac{\partial {P}_{i}}{\partial {x}_{j}}\frac{\partial {P}_{k}}{\partial {x}_{l}}\,{\rm{d}}{x}^{3}+\int {g}_{ijkl}^{{\rm{A}}}\frac{\partial {A}_{i}}{\partial {x}_{j}}\frac{\partial {A}_{k}}{\partial {x}_{l}}\,{\rm{d}}{x}^{3},$$

8 $${F}_{{\rm{electrostatic}}}=\int \left(-\frac{1}{2}{E}_{i}^{{\rm{d}}}{P}_{i}-{E}_{i}^{{\rm{ext}}}{P}_{i}\right){\rm{d}}{x}^{3},$$

9 $${F}_{{\rm{elastic}}}=\int \frac{1}{2}{c}_{ijkl}\left({\varepsilon }_{ij}-{\varepsilon }_{ij}^{0}\right)\left({\varepsilon }_{kl}-{\varepsilon }_{kl}^{0}\right){\rm{d}}{x}^{3},$$

Indices *i*, *j*, *k*, l = 1, 2, 3, 4 indicate components of a vector or a tensor in a Cartesian coordinate. An Einstein summation convention over repeated indices *i*, *j*, *k* and *l* is used. *a*_*i*_, *a*_*ij*_, *a*_*ijk*_ and *a*_*ijkl*_ are the Landau coefficients for ferroelectric polarization. *b*_*i*_, *b*_*ij*_, *b*_*ijk*_ and *b*_*ijkl*_ are the Landau coefficients for antiferroelectric order, **d** is the ferroelectric–antiferroelectric coupling coefficient and **g**^P^ and **g**^A^ are the gradient energy coefficients for ferroelectric polarization and antiferroelectric order, respectively. The Landau free energy function is constructed with local or global minima corresponding to the AFE orthorhombic *Pbcm* phase (that is, **P** = **0** and **A** along ⟨110⟩) and the FE rhombohedral *R3c* phase (that is, **P** along ⟨111⟩ and **A** = **0**) so that only these two phases can be stabilized, thus avoiding the formation of other phases. **E**^d^(**x**) is the depolarization field obtained by solving the electrostatic equilibrium equation at every evolution time step. **c** is the elastic stiffness tensor, **ε**(**x**) is the strain field obtained by solving the mechanical equilibrium equation, and **ε**^0^(**x**) is the eigenstrain field given by ${\varepsilon }_{ij}^{0}={Q}_{ijkl}{P}_{k}{P}_{l}$, with ***Q*** being the electrostrictive coefficient. For details of the mechanical and electrostatic equilibrium equations, please refer to ref. ^53^.

The material constants for NNO used in the phase-field simulations are obtained from refs. ^56–62^. The Landau coefficients for ferroelectric polarization and antiferromagnetic order as well as the ferroelectric–antiferroelectric coupling coefficients are constructed to reproduce the experimental and DFT data of spontaneous polarization, P–E loop, dielectric constant, lattice constant and free energy of the FE and AFE phases^56–58,60,61^, taken as *a*_1_ = −6.5 × 10^7^ J m C^−2^, *a*_11_ = 0.9 × 10^8^ J m^5^ C^−4^, *a*_12_ = 8.0 × 10^8^ J m^5^ C^−4^, *a*_111_ = 3.3 × 10^9^ J m^9^ C^−6^, *a*_112_ = −3.5 × 10^9^ J m^9^ C^−6^, *a*_123_ = −1.0 × 10^9^ J m^9^ C^−6^, *a*_1111_ = −3.1 × 10^10^ J m^13^ C^−8^, *a*_1112_ = 0.2 × 10^10^ J m^13^ C^−8^, *a*_1122_ = 4.2 × 10^10^ J m^13^ C^−8^, *a*_1123_ = −5.0 × 10^10^ J m^13^ C^−8^, *b*_1_ = −6.5 × 10^5^ J m C^−2^, *b*_11_ = 0.9 × 10^8^ J m^5^ C^−4^, *b*_12_ = 7.3 × 10^8^ J m^5^ C^−4^, *b*_111_ = 3.3 × 10^9^ J m^9^ C^−6^, *b*_112_ = −3.5 × 10^9^ J m^9^ C^−6^, *b*_123_ = 25.0 × 10^9^ J m^9^ C^−6^, *b*_1111_ = 3.1 × 10^10^ J m^13^ C^−8^, *b*_1112_ = 0.2 × 10^10^ J m^13^ C^−8^, *b*_1122_ = −4.0 × 10^10^ J m^13^ C^−8^, *b*_1123_ = 15.0 × 10^10^ J m^13^ C^−8^ and *d*_11_ = *d*_12_ = 1.0 × 10^9^ J m^5^ C^−4^. The gradient energy coefficients are obtained from the domain wall width, taken as *g*_11_ = 3.2 × 10^−11^ J m^3^ C^−2^, *g*_12_ = 0 and *g*_44_ = 1.6 × 10^−11^ J m^3^ C^−2^. The background dielectric constant takes a common value across ferroelectric perovskites^62^, ${{\kappa }}_{11}^{{\rm{b}}}=40$. The elastic stiffness is *c*_11_ = 2.30 × 10^11^ J m^−3^, *c*_12_ = 0.90 × 10^11^ J m^−3^ and *c*_44_ = 0.76 × 10^11^ J m^−3^ (ref. ^59^). The electrostrictive coefficients are taken to be isotropic for simplicity with values consistent with our DFT data of the lattice constant differences between *R3c* FE and *Pbcm* AFE phases (Fig. 1c), with *Q*_11_ = 0.160 m^4^ C^−2^, *Q*_12_ = −0.072 m^4^ C^−2^ and *Q*_44_ = 0.084 m^4^ C^−2^.

The phase-field simulations are performed in a two-dimensional simulation system with a total size taken as *l*_1_ × *l*_3_ = 256 nm × 128 nm, which is discretized into an array of 256 × 128 grids. The vertical dimension of the system (with *l*_3_ = 128 nm) along the thickness direction of the film consists of a bottom substrate region of 24 nm, a middle film region of 100 nm and a top vacuum region of 4 nm. An electric potential boundary condition is used at the top and bottom surfaces of the film for the electrostatic equilibrium equation^63^. The piezoelectric performance of the film is modelled by simulating the strain response of the system on applying a given voltage to the top surface of the film while keeping the bottom surface of the film electrically grounded.

## Online content

Any methods, additional references, Nature Portfolio reporting summaries, source data, extended data, supplementary information, acknowledgements, peer review information; details of author contributions and competing interests; and statements of data and code availability are available at 10.1038/s41586-024-07917-9.

## Supplementary information

Supplementary Information

## Figures and Tables

**Extended Data Table 1:**
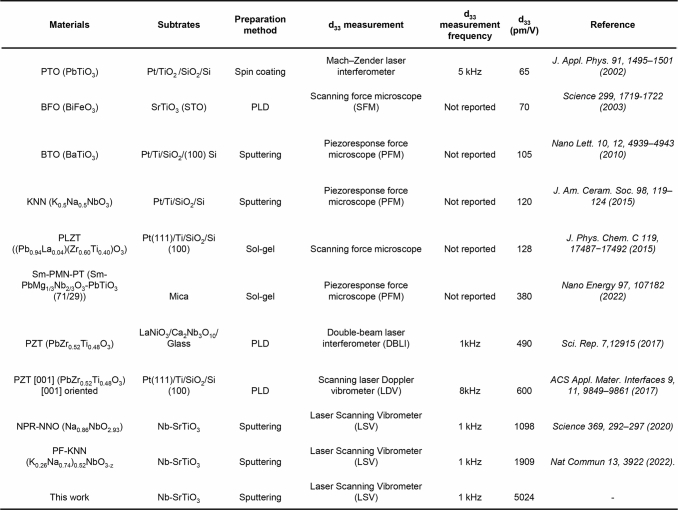
List of Piezoelectric properties of lead and lead-free perovskite thin films^8,17–19,23–27,30^

The effective piezoelectric coefficients of different thin films as well as their fabrication and measurement methods are summarized. These effective piezoelectric coefficients are plotted in the Fig. 1a for comparison.

## Data Availability

The supporting data of this study are available on request from the corresponding authors.
