# Supplementary material for: Ultrahigh electromechanical response from competing ferroic orders
Source: Nature. 2024 Sep 11;633(8031):798–803. doi: 10.1038/s41586-024-07917-9 (PMC11424475; doi:10.1038/s41586-024-07917-9)
Supplement: Supplementary file 1 — Supplementary Information [file 41586_2024_7917_MOESM1_ESM.docx]

**Supplementary Information** for

**Ultrahigh electromechanical response from competing ferroic orders**

Baichen Lin^1,2,⸸^, Khuong Phuong Ong^3,⸸^, Tiannan Yang^4,⸸^, Qibin Zeng^1^, Hui Kim Hui^1^, Zhen Ye^1,5^, Celine Sim^1,2^, Zhihao Yen^2^, Ping Yang^6^, Yanxin Dou^7^, Xiaolong Li^8^, Xingyu Gao^8^, Chee Kiang Ivan Tan^1^, Zhi Shiuh Lim^1^, Shengwei Zeng^1^, Tiancheng Luo^1^, Jinlong Xu^1,9^, Xin Tong^1,10^, Patrick Wen Feng Li^2^, Minqin Ren^7^, Kaiyang Zeng^5^, Chengliang Sun^10^, Seeram Ramakrishna^5^, Mark B. H. Breese^6^, Chris Boothroyd^2,11^, Chengkuo Lee^9^, David J. Singh^12^, Yeng Ming Lam^2,11*^, Huajun Liu^1*^

*^1^Institute of Materials Research and Engineering (IMRE), Agency for Science, Technology and Research (A*STAR), 2 Fusionopolis Way, Innovis #08-03, Singapore 138634, Republic of Singapore*

*^2^School of Materials Science and Engineering, Nanyang Technological University, 50 Nanyang Avenue, Singapore 639798, Republic of Singapore*

*^3^Institute of High Performance Computing (IHPC), A*STAR (Agency for Science, Technology and Research), 1 Fusionopolis Way, #16-16 Connexis, Singapore 138632, Republic of Singapore*

*^4^Interdisciplinary Research Center, School of Mechanical Engineering, Shanghai Jiao Tong University, 800 Dongchuan Road, Shanghai 200240, China*

*^5^Department of Mechanical Engineering, National University of Singapore, 9 Engineering Drive 1, Singapore 117575, Republic of Singapore*

*^6^Singapore Synchrotron Light Source (SSLS), National University of Singapore, 5 Research Link, Singapore 117603, Republic of Singapore*

*^7^Centre for Ion Beam Applications, Department of Physics, National University of Singapore, 2 Science Drive 3, Singapore 117551, Republic of Singapore*

*^8^Shanghai Synchrotron Radiation Facility (SSRF), Shanghai Advanced Research Institute, Chinese Academy of Sciences, Shanghai 200120, China*

*^9^Department of Electrical and Computer Engineering, National University of Singapore, 4 Engineering Drive 3, Singapore 117583, Republic of Singapore*

*^10^Institute of Technological Sciences, Wuhan University, Wuhan 430072, China*

*^11^Facility for Analysis, Characterisation, Testing and Simulation (FACTS), Nanyang Technological University, 50 Nanyang Avenue, Singapore 639798, Republic of Singapore*

*^12^Department of Physics and Astronomy, University of Missouri, Columbia, MO 65211, USA*

*Corresponding authors. Emails: [ymlam@ntu.edu.sg](mailto:ymlam@ntu.edu.sg), [liu_huajun@imre.a-star.edu.sg](mailto:liu_huajun@imre.a-star.edu.sg)

^⸸^ These authors contributed equally to this work

**List of supplementary materials:**

**Supplementary Fig. 1 | The (111) plane-view schematic of STO, NNO (N) phase, and NNO (P) phase.**

**Supplementary Fig. 2 | Composition characterization of the 200 nm-thick NNO films.**

**Supplementary Fig. 3 | Synchrotron-based XRD *2θ-ω* scan of the 200 nm-thick NNO film on Nb-STO (111) substrate.**

**Supplementary Fig. 4 | Estimation of the ratio of N and P phase in the 200 nm-thick NNO films.**

**Supplementary Fig. 5 | STEM and FFT images of the 200 nm-thick NNO films.**

**Supplementary Fig. 6 | Phase boundary derived from the inverse FFT image.**

**Supplementary Fig. 7 | Ferroelectric P-E hysteresis loop of the 200 nm-thick NNO films.**

**Supplementary Fig. 8 | Piezoresponse force mapping characterizations of the 200 nm-thick NNO films.**

**Supplementary Fig. 9 | Electromechanical response of the 200 nm-thick NNO films.**

**Supplementary Fig. 10 | TEM cross-sectional images of the post-vibrometer measurement samples.**

**Supplementary Fig. 11 | Spatial distribution of the piezoelectric coefficient d_33_ at E = 300 kV/cm, from phase-field simulations.**

**Supplementary Fig. 12 | Simulated polarizations of Nb and Na atoms in the NNO P phase using DFT.**

**Supplementary Fig. 13 | Schematics of Landau potential energy landscape.**

**
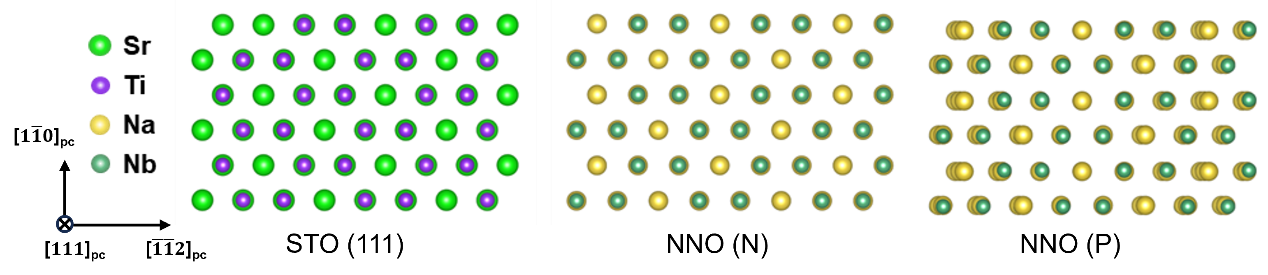
**

**Supplementary Fig. 1 | The (111) plane-view schematic of STO, NNO (N) phase, and NNO (P) phase.** The atomic structures of (111) of STO, NNO (N), and NNO (P) depict that the N phase is of the same arrangement of atoms with STO, indicating a priority of epitaxial growth compared with the P phase.

**
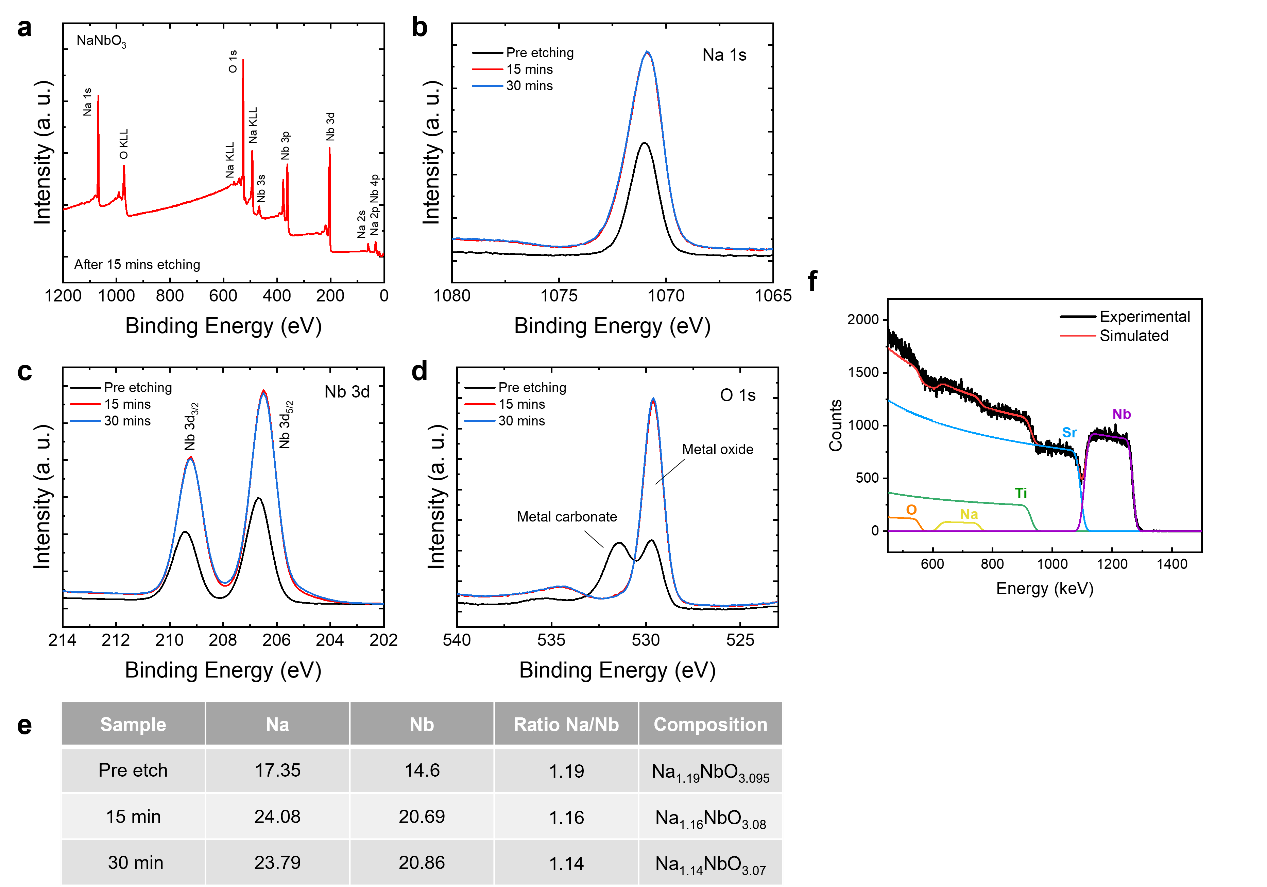
**

**Supplementary Fig. 2 | Composition characterization of the 200 nm-thick NNO films.** (a) XPS survey spectra of NNO film with 15-min surface etching. (b) Na *1s*, (c) Nb *3d*, and (d) O *1s* XPS spectra of the NNO film before etching (black), after 15-min (red) and 30-min (blue) etching. (e) Calculated composition of the NNO film under different etching time. (f) Rutherford backscatter spectrum of the NNO film. The composition is Na_1.16_NbO_3.08_ based on the fitting result, which is consistent with the XPS result.

**
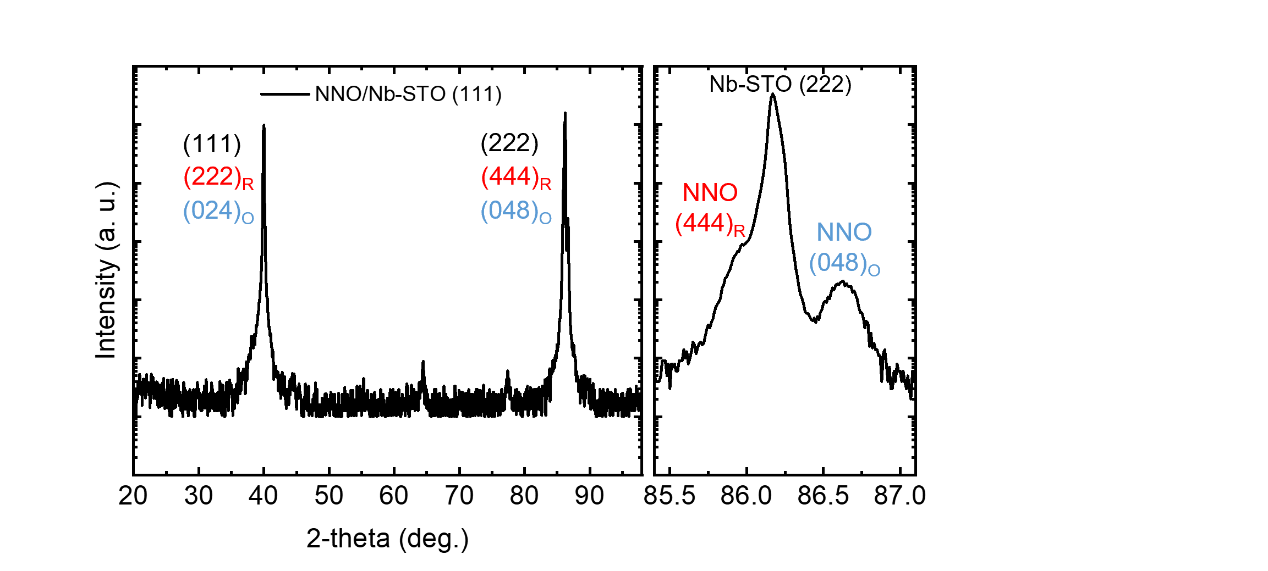
**

**Supplementary Fig. 3 | Synchrotron-based XRD *2θ-ω* scan of the 200 nm-thick NNO film on Nb-STO (111) substrate.** The NNO film is epitaxially grown on the Nb-STO (111) substrate. As shown in the zoom-in pattern, the left peak refers to the (444)_R_ of the N phase, while the right peak refers to the (048)_O_ of the P phase. Note that both of them could be regarded as (222) based on pseudocubic notation.

**
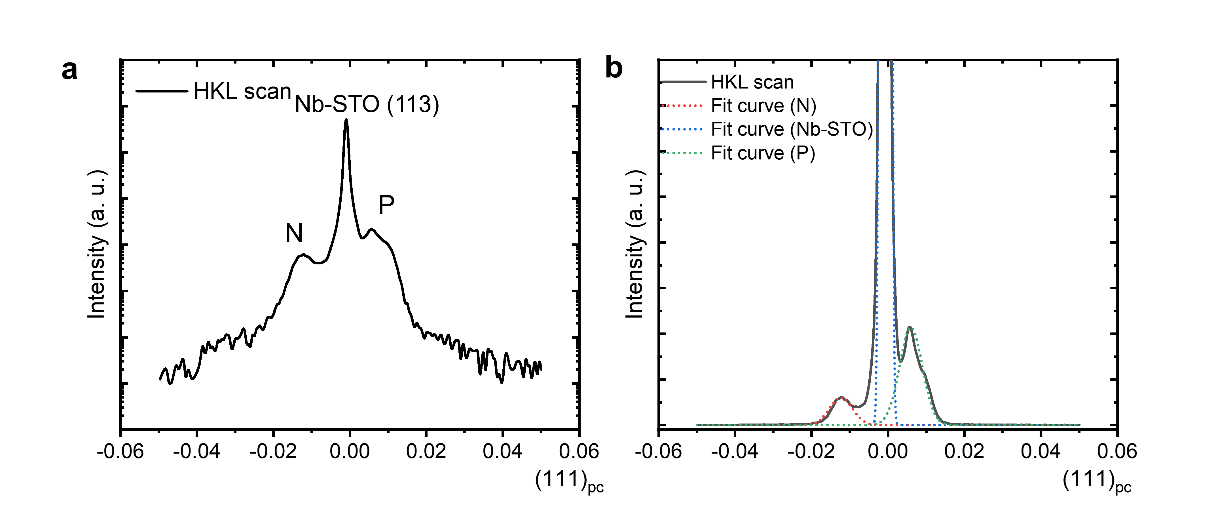
**

**Supplementary Fig. 4 | Estimation of the ratio of N and P phase in the 200 nm-thick NNO films.** (a) HKL scan of (113) reflection along (111) direction. (b) Fitting diffraction pattern in terms of Gaussian curves. By integrating the area of each fitting peak, the ratio of the N and P phases is about 1: 3.

**
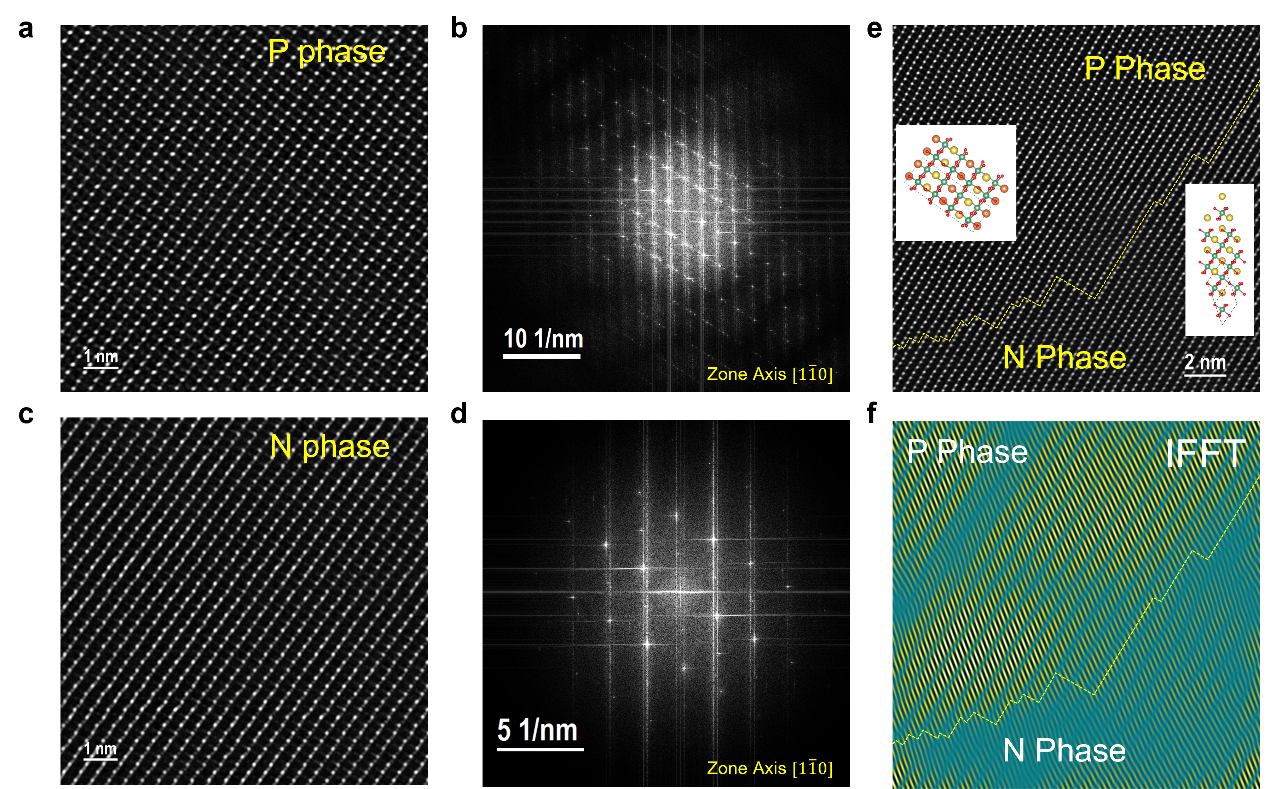
**

**Supplementary Fig. 5 | STEM and FFT images of the 200 nm-thick NNO films.** (a, c) STEM images of (a) P phase and (c) N phase and (b, d) their corresponding FFT patterns. (e) Phase boundary image taken by STEM and (f) its corresponding inversed FFT image.

**
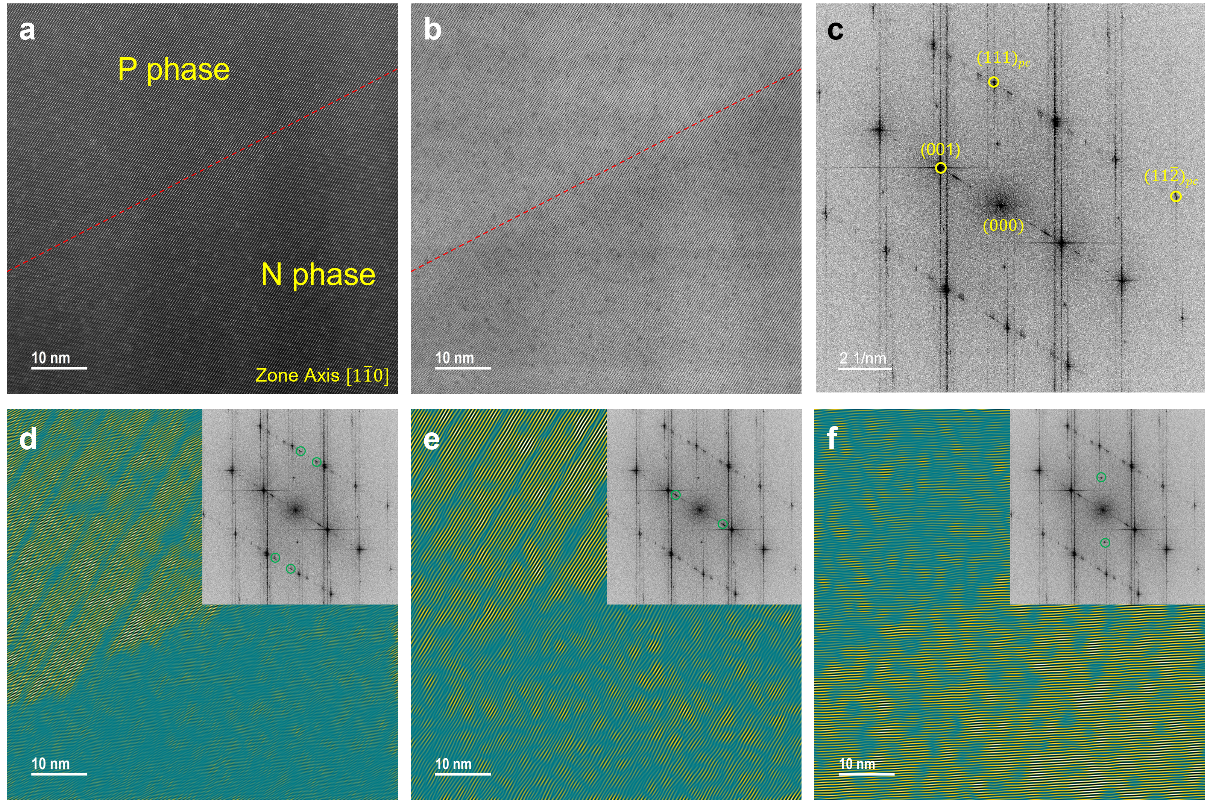
**

**Supplementary Fig. 6 | Phase boundary derived from the inverse FFT image.** (a) HAADF, (b) BF images and (c) FFT pattern of the NNO film taken by STEM. (d-f) IFFT images were created by choosing different fingerprint-diffraction spots in the FFT, indicating a clear phase boundary between the P and N phases and structural stripes in the P phase.

**
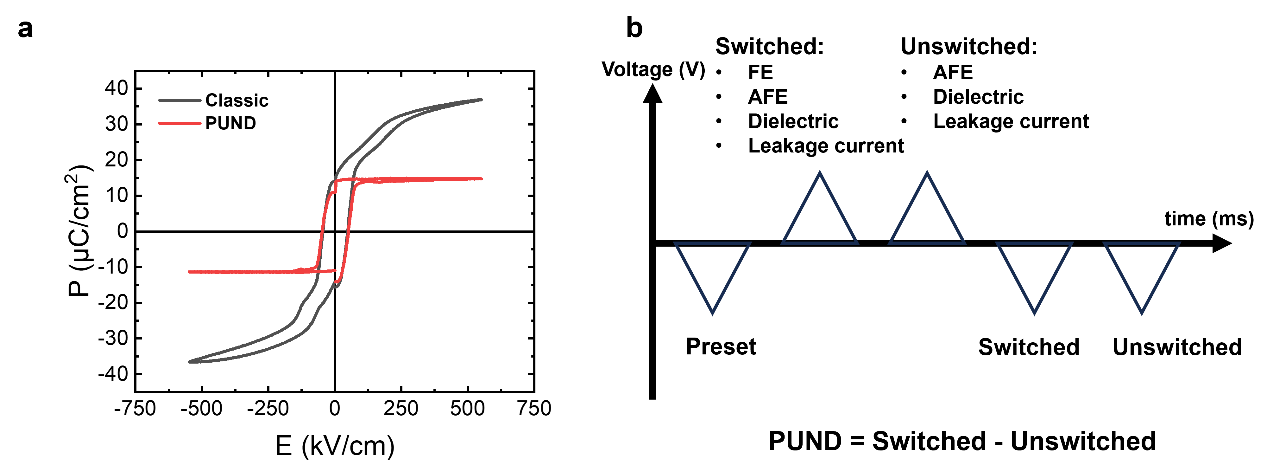
**

**Supplementary Fig. 7 | Ferroelectric P-E hysteresis loop of the 200 nm-thick NNO films.** (a) Conventional P-E loop and PUND (positive up negative down) results. (b) Schematic of the ferroelectric PUND measurement. The AFE phase has no contribution in the PUND loop as its remnant polarization is zero. The remnant polarization is about 14 μC/cm^2^. The measurements were performed using symmetrical La_0.7_Sr_0.3_MnO_3_ (LSMO) electrodes.

**
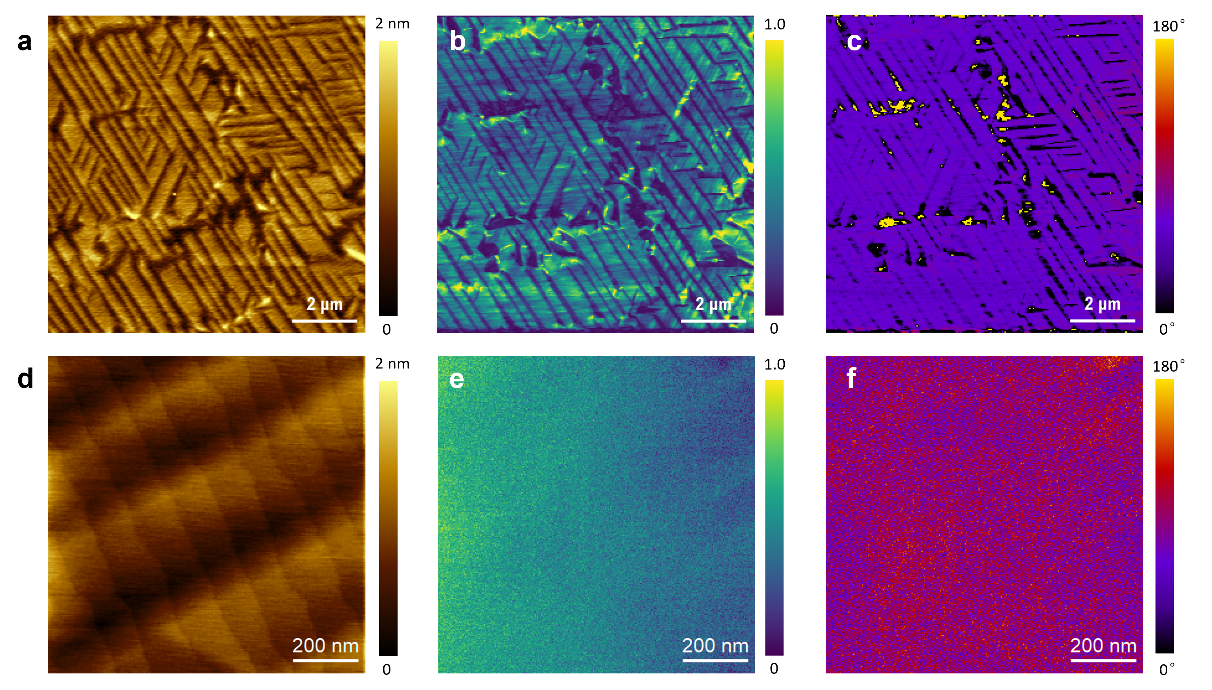
**

**Supplementary Fig. 8 | Piezoresponse force mapping characterizations of the 200 nm-thick NNO films.** (a) Topography, (b) amplitude, and (c) phase images based on out-of-plane PFM. (d) Topography, (e) amplitude, and (f) phase images based on in-plane PFM.

**
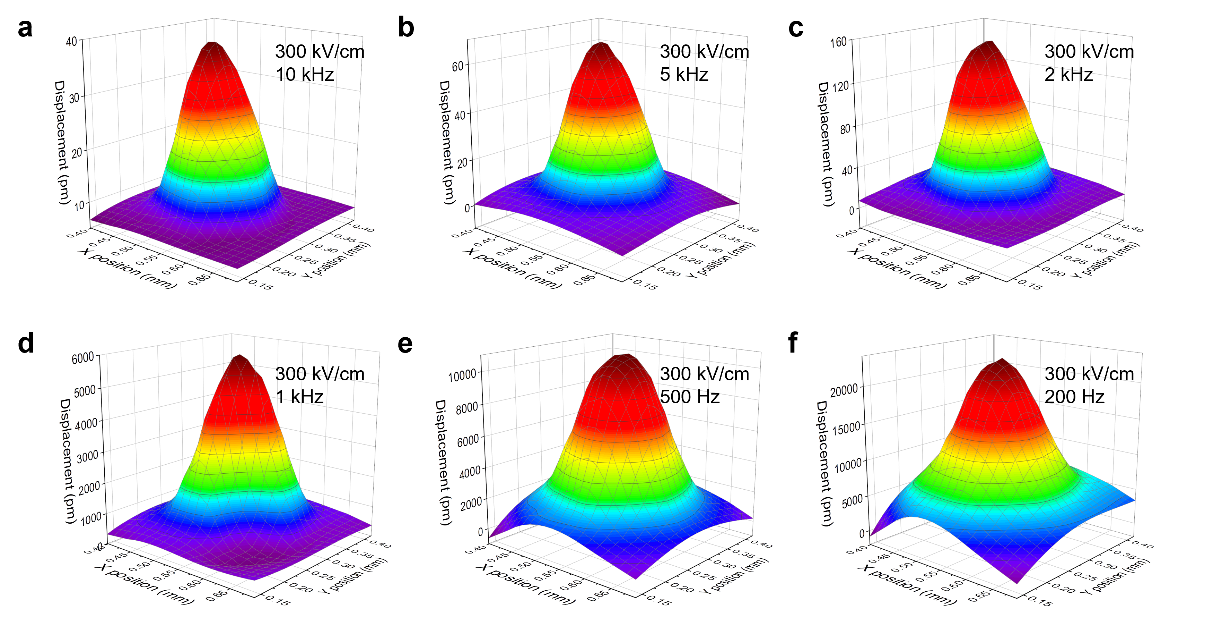
**

**Supplementary Fig. 9 | Electromechanical response of the 200 nm-thick NNO films.** Frequency-dependent surface displacement measured by laser Doppler vibrometer with an a.c. voltage of 1 V under an external electric field of 300 KV/cm. (a) 10 kHz. (b) 5 kHz. (c) 2 kHz. (d) 1 kHz. (e) 500 Hz. (f) 200 Hz.

**
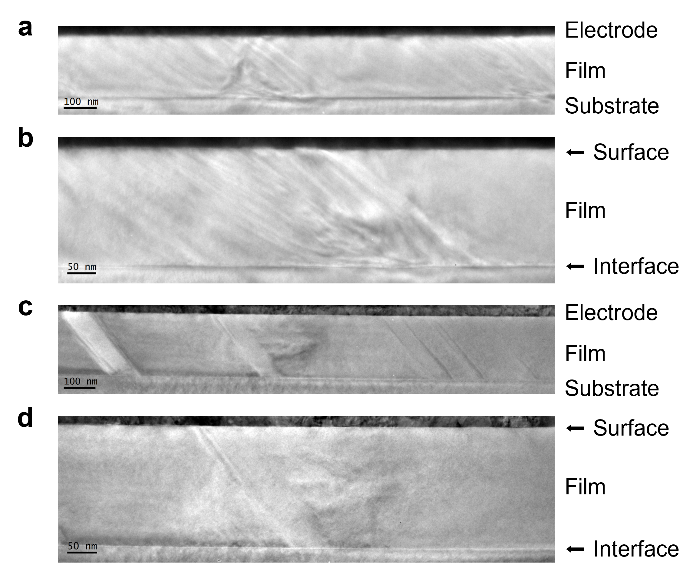
**

**Supplementary Fig. 10 | TEM cross-sectional images of the post-vibrometer measurement samples.** Sample 1 and Sample 2 were fabricated from different areas but under the same electrode. (a) Sample 1. Scale bar: 100 nm. (b) Sample 1. Scale bar: 50 nm. (c) Sample 2. Scale bar: 100 nm. (d) Sample 2. Scale bar: 50 nm.

**Supplementary Fig. 11 | Spatial distribution of the piezoelectric coefficient d_33_ at E = 300 kV/cm, from phase-field simulations.**

**
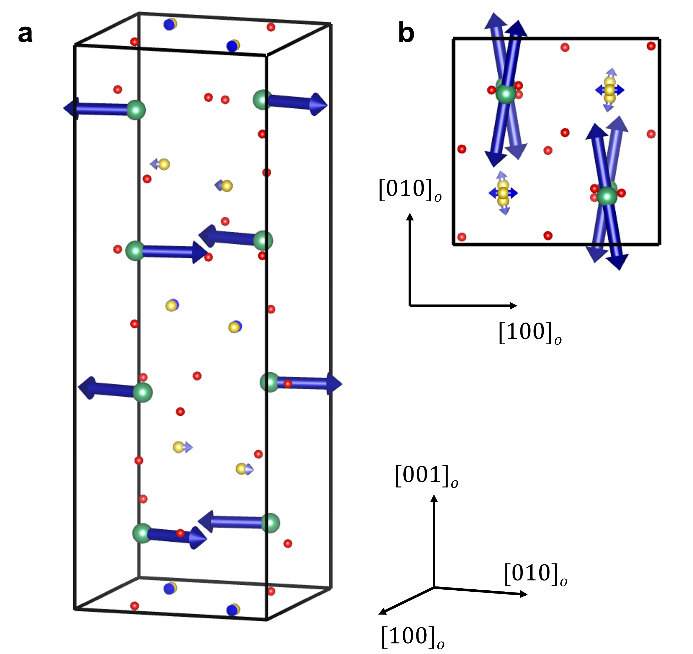
**

**Supplementary Fig. 12 | Simulated polarizations of Nb and Na atoms in the NNO P phase using DFT.** (a) Calculated *Pbcm* structure of NNO thin film. The dark and light blue vectors indicate the polarization directions of Nb and Na atoms. (b) Projection view of the calculated structure from [001]_o_.


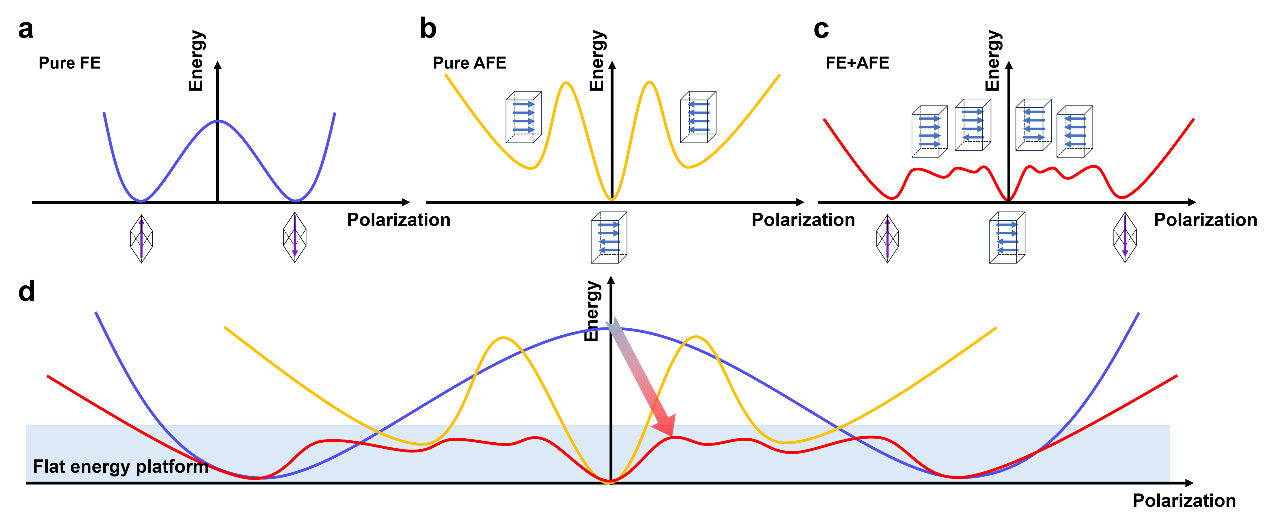


**Supplementary Fig. 13 | Extended Data Fig. 22 | Schematics of Landau potential energy landscape.** (a) Pure FE NNO N phase. (b) Pure AFE NNO P phase. (c) FE N phase and AFE P phase mixtured. The ↑↑↑↓ polarization states occur possibly at the phase boundary. (d) Flat energy platform induced by the competition of FE and AFE orders.
